# Layered Alkali Metal Titanate with the Staging Structure and Superior Electrochemical Performance

**DOI:** 10.1021/acs.inorgchem.5c01712

**Published:** 2025-08-04

**Authors:** Aranee Pleng Teepakakorn, Tomohiro Tanaka, Nobuyuki Sakai, Yasuo Ebina, Takayuki Kikuchi, Renzhi Ma, Makoto Ogawa, Takayoshi Sasaki

**Affiliations:** † Research Center for Materials Nanoarchitectonics (MANA), 52747 National Institute for Materials Science (NIMS), 1-1 Namiki, Tsukuba, Ibaraki 305-0044, Japan; ‡ School of Energy Science and Engineering, 423058 Vidyasirimedhi Institute of Science and Technology (VISTEC), 555 Moo 1 Payupnai, Wangchan, Rayong 21210, Thailand

## Abstract

We systematically explored the formation range of α-NaFeO_2_-type layered titanate and its ion-exchange behaviors and electrochemical performance. The layered sodium titanate of Na_*x*_Ti_1–*x*/3_Li_*x*/3_O_2_ at *x* = 0.68–0.70 was synthesized by the solid-state reaction at 900 °C. The titanate is composed of coplanar host layers with the α-NaFeO_2_-type structure. The interlayer Na^+^ ions underwent a facile exchange with other alkali metal ions in aqueous solutions at 80 °C, accompanied by concurrent exchange with proton/oxonium ions. Powder X-ray diffraction data on the products and their Rietveld refinement revealed the formation of a unique staging-structured titanate, in which the interlayer galleries are alternately occupied by incoming alkali metal ions and oxonium ions. The electrochemical intercalation–deintercalation properties for Li^+^ and Na^+^ ion storage were examined on the pristine sodium titanate and its ion-exchanged derivatives. The staging structure was found to provide superior electrochemical performance, which may be due to the rather open nature of the interlayer galleries, providing abundant sites for Li^+^ and Na^+^ ions.

## Graphical abstract

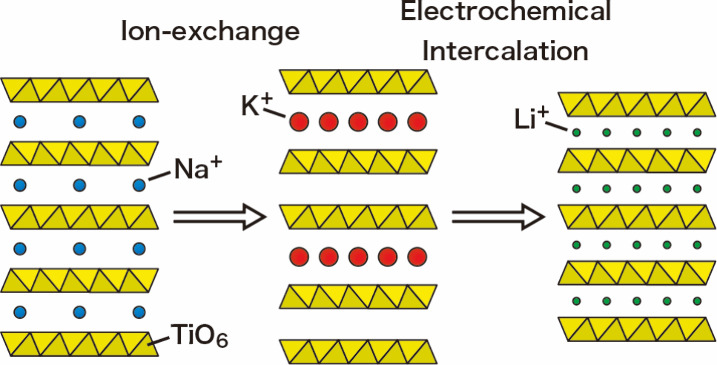

## 1 Introduction

Considerable attention has been paid to layered transition metal oxides for their useful functions, such as photocatalytic and dielectric properties.^1–7^ Layered alkali metal titanates with various structures are known, as illustrated in Figure 1. A series of titanates, such as Na_2_Ti_3_O_7_, K_2_Ti_4_O_9_, and Cs_2_Ti_5_O_11_, have corrugated host layers of edge-shared TiO_6_ octahedra, which are stepped via corner-sharing every three, four, and five octahedra, respectively.^8–10^ A class of lepidocrocite-type (γ-FeOOH) layered titanates with the general formula of A_*x*_Ti_2–*y*_M_*y*_O_4_ (where A = K, Rb, Cs and M = vacancy, Li, Mg, Ni, Co, Zn, Cu, Fe­(III), Mn­(III))^11–14^ can be regarded as their relatives with the infinite stepping width. The host layers of these compounds are negatively charged, and charge-compensating alkali metal ions are accommodated between them. The interlayer cations undergo facile ion-exchange reactions under ambient conditions.^15–18^ This reactivity enables the intercalation of various organic and inorganic guest species to yield a range of nanocomposites and hybrid materials. Furthermore, the titanates can be exfoliated into colloidal individual layers via swelling, typically with some amines and organoammonium ions.^19–21^ The resulting 2D oxide nanosheets can be organized as a building block into precisely designed nanostructured materials. Functionalization of the titanates through these processes has been studied extensively.^5,7,22^

**Figure 1:**
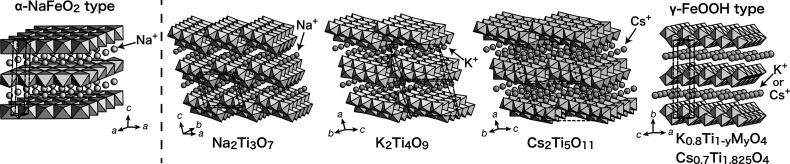
Crystal structures of various types of layered titanates. The unit cell is indicated by the broken line.

There is another class of layered alkali metal titanates that are characterized by coplanar host layers based on a hexagonal linkage of TiO_6_ octahedra (Figure 1). The titanates of the α-NaFeO_2_-type structure have been less investigated compared with the other compounds described above. The layered titanate based on the α-NaFeO_2_-type structure was first obtained by the electrochemical deintercalation of Na^+^ from NaTiO_2_ to form Na_*x*_Ti^4+^_1–*x*_Ti^3+^_*x*_O_2_.^23,24^ Isomorphous substitution of Ti^4+^ in the sheet with Co^2+^, Ni^2+^, and Li^+^ has been reported to yield Na_2/3_Co_1/3_Ti_2/3_O_2_, Na_*x*_Ni_*x*/2_Ti_1–*x*/2_O_2_ (0.6 < *x* < 0.66), and Na_0.66_Li_0.22_Ti_0.78_O_2_ through solid-state synthesis.^25–27^ Their chemical reactivities, such as ion exchange and redox intercalation, have not been explored in depth, except for electrochemical reactivities toward possible application as a negative electrode for sodium-ion batteries.

In the present study, the α-NaFeO_2_-type layered alkali metal titanate of Na_*x*_Ti_1–*x*/3_Li_*x*/3_O_2_ (*x* ∼ 0.68) was synthesized by solid-state calcination of starting reagents Na_2_CO_3_, Li_2_CO_3_, and TiO_2_. The ion-exchange behaviors of Na_*x*_Ti_1–*x*/3_Li_*x*/3_O_2_ with the alkali metal ions (Li^+^, Na^+^, K^+^, Rb^+^, and Cs^+^) from aqueous solutions were examined at 80 °C. We found that the ion exchange proceeded by forming a unique staging structure composed of alternately occupied and unoccupied interlayer galleries. The titanate of Na_*x*_Ti_1–*x*/3_Li_*x*/3_O_2_ and its ion-exchanged phases showed electrochemical intercalation/deintercalation properties for the storage of Li^+^ and Na^+^ ions.

## 2 Experimental Section

### 2.1 Chemicals and Materials

Titanium dioxide (rutile, 99.99%), lithium carbonate (99.99%), sodium carbonate (99.99%), rubidium chloride (99.9%), and cesium chloride (99.99%) were obtained from Rare Metallic, Japan, and used as received. Sodium chloride (99.5%) and potassium chloride (99.5%) were obtained from Kanto Chemical, Japan. Lithium chloride (99.0%) was purchased from Fujifilm Wako Pure Chemical, Japan. Acetylene black (HS100) was purchased from Denka, Japan. Polyvinylidene fluoride suspension (12 wt %, PVDF) was obtained from Kureha, Japan. *N*-Methyl-2-pyrrolidone (NMP, 99.5%) was purchased from Kishida Chemical, Japan. A Whatman glass fiber filter (thickness 200–300 μm) was obtained from Merck.

### 2.2 Synthesis of Layered Sodium Titanate

Reagents of Na_2_CO_3_, Li_2_CO_3_, and TiO_2_ were intimately mixed at a molar ratio of *x*/2:*x*/6:(1–*x*/3) for the composition Na_*x*_Ti_1–*x*/3_Li_*x*/3_O_2_. The explored *x* value was 0.60 ≤ *x* ≤ 0.80. The mixture was placed in a Pt crucible and heated at 900 °C for 30 min for decarbonation. After cooling, the powder was ground and then heated at 900 °C for 24 h. The obtained sample, Na_0.68_Ti_0.77_Li_0.23_O_2_, referred to as NTLO, was used in the following experiments to explore its chemical reactivities.

### 2.3 Ion Exchange of Layered Sodium Titanate with Alkali Metal Ions

Ion-exchange experiments were carried out by equilibrating 0.2 g of NTLO with 20 cm^3^ of an aqueous solution of alkali metal chloride (0.5 M) at 80 °C for 24 h. The treatment was repeated 2 times by decanting the solution with the fresh one. The products were collected by filtration, washed with deionized water several times, and then dried overnight at room temperature. The samples after the ion exchange of NTLO with alkali metal ions, Li^+^, Na^+^, K^+^, Rb^+^, and Cs^+^, are designated as Li-NTLO, Na-NTLO, K-NTLO, Rb-NTLO, and Cs-NTLO, respectively. The ion exchange for Li^+^ and Na^+^ was also examined under the same conditions with K-NTLO, and the obtained samples are referred to as Li-KNTLO and Na-KNTLO, respectively.

### 2.4 Electrochemical Studies

The electrode materials (NTLO and Na-KNTLO), acetylene black, and PVDF were mixed in a weight ratio of 88:8:4 in NMP using a mixer (AR-100, Thinky). The obtained slurry was spread onto a Cu foil using blade coating and dried at 80 °C for NTLO and at 50 °C for Na-KNTLO under N_2_ gas overnight, and it was then pressed in order to closely pack the electrode composite on the Cu foil and dried at 110 °C for NTLO and at 50 °C for Na-KNTLO under vacuum for 15 h. The lower drying temperature applied to the Na-KNTLO electrodes was intended to suppress collapse of the staging structure. The loading amount of the electrode materials was 7–12 mg/cm^2^. The resulting electrodes were used to assemble a CR2032 coin-type cell with Li or Na foil as the counter electrode and a glass fiber filter (thickness 200–300 μm) as the separator in a dry atmosphere (<0.2 ppm of H_2_O). LiPF_6_ (1 M) in ethylene carbonate (EC)/diethyl carbonate (DEC) (3:7 in v/v) and 1 M sodium bis­(trifluoromethanesulfonyl)­imide (NaTFSI) in EC/dimethyl carbonate (DMC) (1:1 in v/v) were used as electrolytes for Li^+^- and Na^+^-ion batteries, respectively. The intercalation/deintercalation studies were carried out using a charge/discharge unit (HJ1001SD8, Hokuto Denko, Japan) at room temperature.

### 2.5 Characterizations

Powder X-ray diffraction (XRD) data were recorded by using a Rigaku ULTIMA IV diffractometer with monochromatic Cu Kα radiation (λ = 0.15405 nm). The high-resolution data for NTLO and K-NTLO were collected at BL02B1 of the SPring-8 synchrotron radiation facility (Hyogo, Japan) under the agreement from the Japan Synchrotron Radiation Research Institute (JASRI) and analyzed by Rietveld refinement with the program RIETAN-2000.^28^ Scanning electron microscopy (SEM) observations were performed with JEOL, JSM-6010LA. For chemical analysis of the titanates, a weighed amount (∼0.1 g) of the sample was dissolved with a mixed acid solution (H_2_SO_4_ + HF) at 135 °C for 16 h. Then, metal ion contents were determined by inductively coupled plasma-optical emission spectrometry (ICP-OES, Hitachi, SPS3520UV-DD) after appropriate dilution with water. Thermogravimetric differential thermal analysis (TG-DTA) was performed with the instrument of Rigaku, TG-8120, at a heating rate of 10 °C/min in the temperature range of 25–1000 °C.

## 3 Results and Discussion

### 3.1 Synthesis and Structural Characterizations

An intimate mixture of Na_2_CO_3_, Li_2_CO_3_, and TiO_2_ for the composition of Na_*x*_Ti_1–*x*/3_Li_*x*/3_O_2_ with the *x* value of 0.6–0.8 was heated at 900 °C. The obtained samples were composed of several tens of micrometer-sized particles with a granular to platy shape (Figure S1). Powder XRD data on the products in the range 0.68 ≤ *x* ≤ 0.70 could be indexed in terms of the hexagonal structure with unit cell dimensions of *a* ∼ 0.296 and *c* ∼ 1.11 nm (Figure 2), confirming the single-phase formation of the α-NaFeO_2_-type layered titanate. On the other hand, impurity phases, such as Na_2_Ti_6_O_13_ and Li_4_Ti_5_O_12_, coexisted with the target compound outside this range.

**Figure 2:**
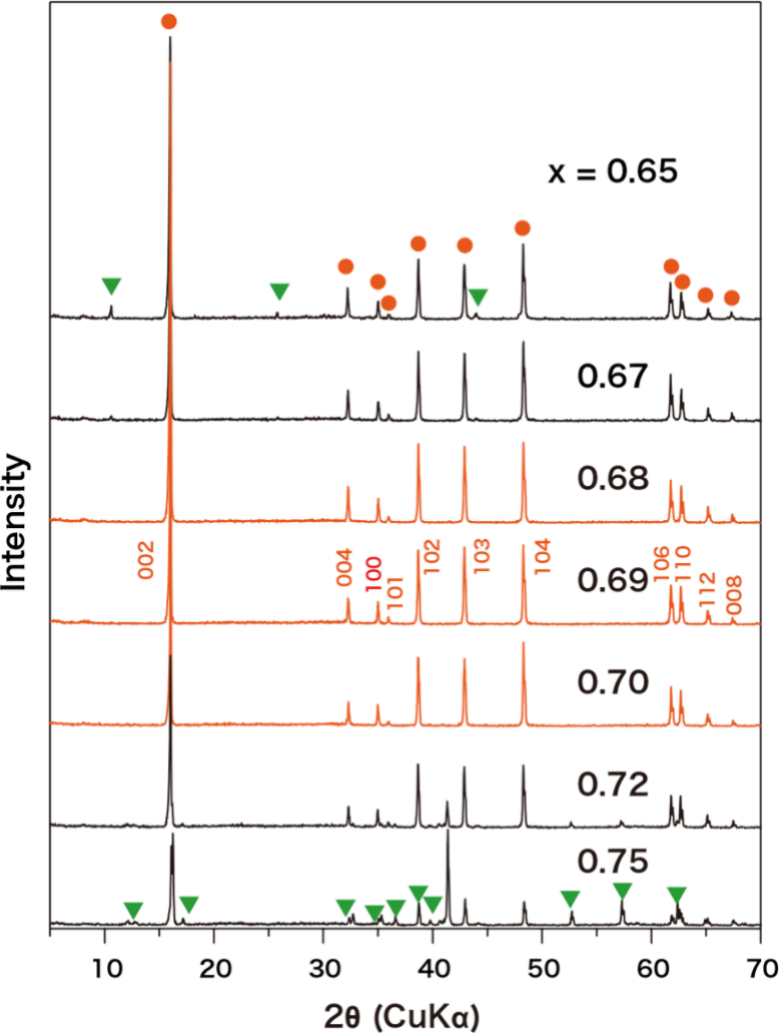
XRD patterns of calcined samples for Na_*x*_Ti_1–*x*/3_Li_*x*/3_O_2_ with the *x* value of 0.65–0.75. The peaks with orange circles are attributable to the titanate with the α-NaFeO_2_-type structure, while those with green triangles are from impurity phases.

Crystal structure of NTLO was refined on its high-resolution synchrotron XRD data based on a model with the α-NaFeO_2_-type layered structure (space group: *P*6_3_/*mmc* (No. 194)). As shown in Figure S2, the refinement led to satisfactory fitting with the residual indices *R*_wp_ = 0.0798, *R*_p_ = 0.0620, *R*_I_ = 0.0392, *R*_F_ = 0.027, and *s* = 1.952. The atomic positional parameters are listed in Table S1. The results are comparable to previous reports.^27^ As illustrated in Figure 1, MO_6_ octahedra are joined via edge-sharing to form the coplanar host layer of Ti_0.77_Li_0.23_O_2_. It is known that oxide layers in the α-NaFeO_2_-type architecture are stacked in various sequences to provide suitable coordination environments for interlayer cations.^29,30^ Two of the most typical stacking modes are P2 and O3, where P and O represent the trigonal prismatic and octahedral coordination, respectively, while 2 and 3 indicate the number of oxide layers in the unit cell. The NTLO in this study adopts the P2 structure. The interlayer gallery is 0.556 nm high (= *c*/2), accommodating Na^+^ ions at trigonal prismatic sites (2b and 2d). Their distribution in these sites was refined, revealing the preferred occupancy at the 2d site. This tendency has been reported for other isomorphous-layered metal oxides, e.g., Na_0.74_CoO_2_.^31^ The interatomic distance between the metal site in the host layer and the interlayer 2b site is closer, leading to a less stable accommodation.

### 3.2 Ion-Exchange Behaviors

Ion-exchange behaviors were examined by treating NTLO with aqueous solutions of alkali metal chlorides at 80 °C. SEM observations indicate that the particle size and platelet morphology remained virtually unchanged after the treatment, suggesting a topotactic reaction process (Figure S3). The chemical composition of the samples after the ion-exchange reaction is given in Table 1. Na^+^ ions were nearly absent when NTLO was treated with aqueous solutions of KCl, RbCl, and CsCl. It should be pointed out that the amount of incoming K^+^, Rb^+^, and Cs^+^ ions is much less than that of Na^+^ ions originally accommodated, indicating that stoichiometric ion exchange between alkali metal ions did not take place. This difference may be explained by concurrent proton exchange. Based on ignition loss results, the proton contents were estimated, as indicated in Table 1. The chemical formulas of K-NTLO, Rb-NTLO, and Cs-NTLO indicate that approximately half of the protons is accommodated in the form of oxonium ions, and another half is in the form of H^+^. The FT-IR spectrum of ion-exchanged phases (Figure S4) is consistent with the assignment above. A broad absorption band at 3600–2700 cm^–1^ and a rather sharp peak at 1600 cm^–1^ are attributable to stretching and bending modes of water molecules, respectively, indicating the presence of oxonium ions. On the other hand, the strong absorption at 930 cm^–1^ is characteristic of the hydroxyl group, which may be formed via bonding of a proton to the oxygen atom on the host layer. A similar feature was observed in protonated layered titanates, such as H_2_Ti_4_O_9_·1.3H_2_O and H_2_Ti_5_O_11_·3H_2_O,^16,17,32^ which contain oxonium ions and hydroxyl groups. Another noteworthy point is that the Li content remained virtually unchanged, indicating that Li^+^ ions in the host layer were not involved in the ion-exchange reaction. Figure 3 shows powder XRD data of the ion-exchanged products. All the peaks could be indexed in terms of a hexagonal structure, and the refined unit cell parameters are summarized in Table 1. Different from the parent compound (NTLO) of Na_0.68_Ti_0.77_Li_0.23_O_2_, basal peaks having odd indices appeared, and their intensity is relatively weak compared with those with even indices. These features suggest that the layered titanate of NTLO underwent some structural change upon ion exchange, as will be discussed below in depth.

**Figure 3:**
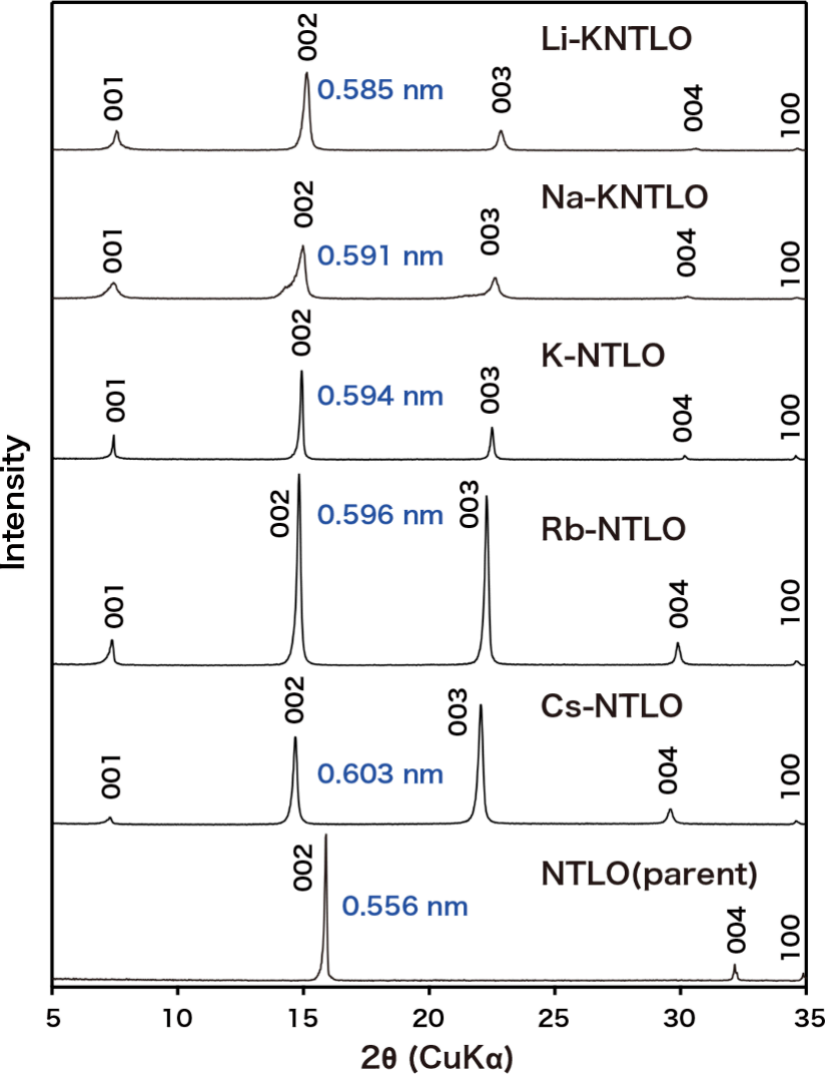
XRD patterns of NTLO and its ion-exchanged phases with alkali metal ions. Values in blue represent the *d*-spacing of the 002 peak.

**Table 1:** Chemical Composition and Unit Cell Dimensions of Ion-Exchanged Phases

sample	composition	lattice constants (nm)	
		*a*	*c*
NTLO	Na_0.68_Ti_0.77_Li_0.23_O_2_	0.29611(4)	1.1113(2)
Li-KNTLO^a^	Li_0.13_H_0.56_Ti_0.77_Li_0.23_O_2_·0.39H_2_O	0.29879(4)	1.1674(2)
Na-KNTLO	Na_0.17_H_0.58_Ti_0.77_Li_0.17_O_2_·0.36H_2_O	0.29938(4)	1.1811(2)
K-NTLO	K_0.15_H_0.56_Ti_0.77_Li_0.19_O_2_·0.27H_2_O	0.29877(5)	1.1818(3)
Rb-NTLO	Rb_0.13_H_0.55_Na_0.02_Ti_0.77_Li_0.21_O_2_·0.25H_2_O	0.29875(4)	1.1928(2)
Cs-NTLO	Cs_0.12_H_0.56_Na_0.02_Ti_0.77_Li_0.21_ O_2_·0.25H_2_O	0.29894(4)	1.2034(3)

a The Li content in the host layer is assumed to be maintained during the ion-exchange process.

On the other hand, treatment with the LiCl solution did not produce a material comparable to ion-exchanged phases with K^+^, Rb^+^, and Cs^+^ ions but yielded a poorly crystalline sample, showing rather broad diffraction peaks (Figure S5a). Chemical analysis revealed that ∼2/3 of Na^+^ ions were replaced with Li^+^ ions, and the proton exchange was not significant. The sample treated with the NaCl solution showed the XRD pattern for pristine NTLO accompanied by some minor byproducts (Figure S5b). In contrast, when K-NTLO was treated with aqueous solutions of LiCl and NaCl, an ion-exchange reaction took place in a similar way to the process of K^+^, Rb^+^, and Cs^+^ ions from NTLO. Powder XRD data after the reaction show the formation of such a unique phase (Figure 3). The chemical compositions were comparable to those for K-, Rb-, and Cs-exchanged phases, except for higher degrees of hydration (Table 1).

### 3.3 Staging Structure of Ion-Exchanged Materials

As described above, after the ion-exchange process, 00*l* basal diffraction peaks (*l* = 2*n* + 1) appeared, suggesting a change in the stacking periodicity along the *c*-axis. There are two possibilities to account for this feature: (i) different occupancies of guests in adjacent interlayer galleries and (ii) noneven displacement of the host layers along the *c*-axis. Because no systematic extinction of diffraction peaks was recognized, five space groups, such as P6̅m2 , P6̅2m , *P*6*mm*, *P*622, and *P*6/*mmm*, are possible. After considerations, we found that a reasonable structure model can be constructed according to the space group P6̅m2 . Then, the structure analysis was conducted for the K^+^ ion-exchanged phase, K-NTLO, as the representative sample. The refinement yielded a reasonable convergence with the residual indices of *R*_wp_ = 0.0597, *R*_p_ = 0.0442, *R*_I_ = 0.0286, *R*_F_ = 0.0159, and *s* = 1.590 (Figure 4). The structural parameters are summarized in Table 2, and the refined structure is illustrated in Figure 5. The host layers are stacked along the *c*-axis with alternate repeating spacings of 0.700 and 0.482 nm. The neighboring layers glide with respect to each other along the *a*-axis by *a*/2, generating a trigonal prismatic site for interlayer guest species. The wider interlayer galleries accommodate guest species, such as K^+^ and H_3_O^+^ ions, while the narrower galleries are empty. The large thermal parameter may reflect from widely distributed position of these guest species, in addition to expressing their thermal vibration. As discussed above, this phase contains protons, as well as H_3_O^+^ ions. The position of protons is not available because the structure refinement is based on the XRD data.

**Figure 4:**
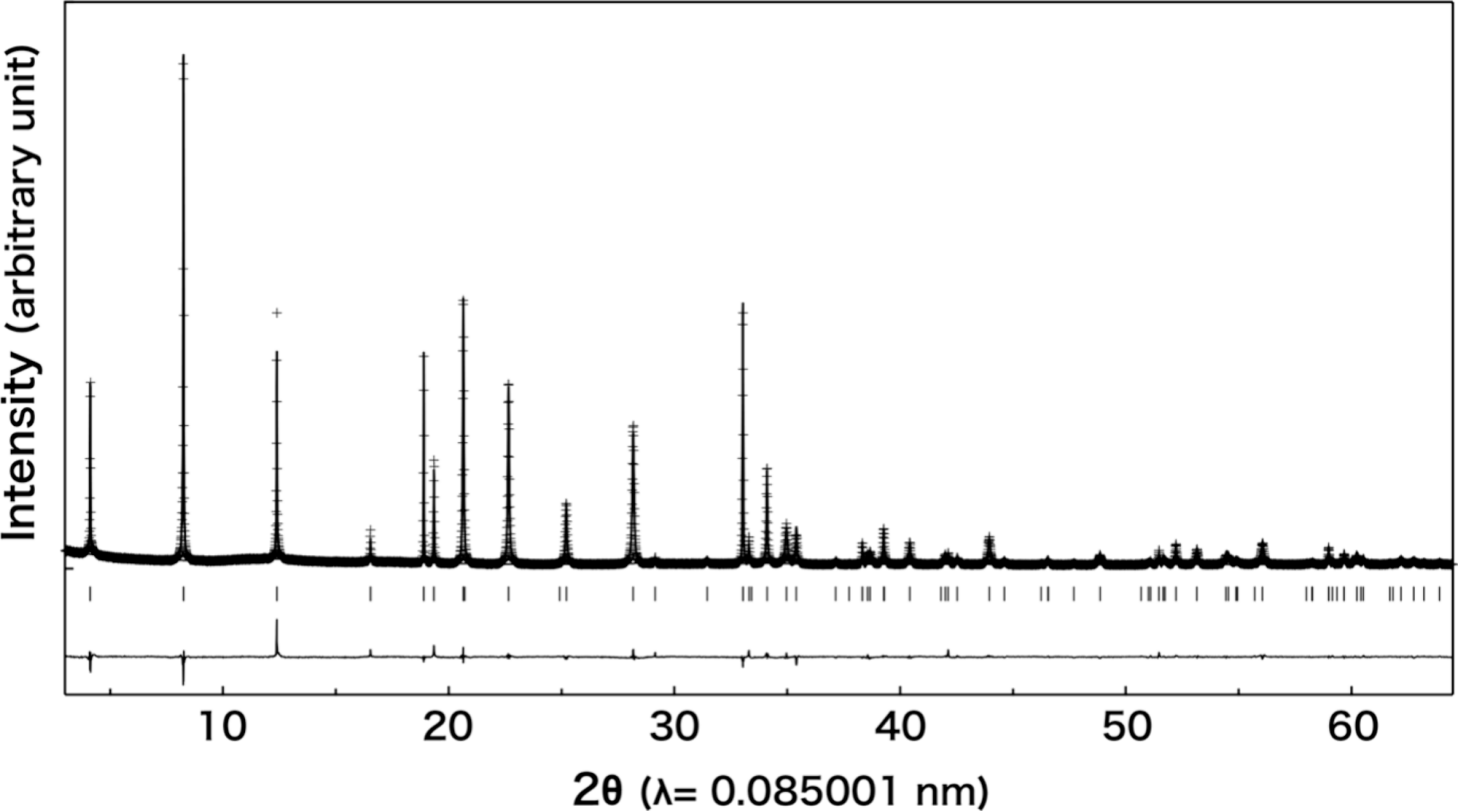
Rietveld fitting of synchrotron X-ray diffraction data for K-NTLO. Observed and calculated profiles are denoted by dotted and solid lines, respectively. The differences between them and locations of reflections are indicated at the bottom.

**Table 2:** Structural Parameters for K-NTLO^a^

atom	position	occupancy^d^	*x*	*y*	*z*	*B*_eq_ (×10^–2^ nm^2^)
*G*1^b^	1b	0.120(1)	0	0	1/2	18.7(1)
*G*2^b^	1f	0.244	2/3	1/3	1/2	15.9
*M* ^c^	2g	1	0	0	0.20393(5)	0.60(1)
O1	2h	1	1/3	2/3	0.2864(1)	0.98(4)
O2	2i	1	2/3	1/3	0.1128(1)	1.02(3)

a Hexagonal, P6̅m2 (No. 187), *a* = 0.299035(1) nm, *c* = 1.18259(1) nm.

b *G* = 0.15 K^+^ + 0.27 H_3_O^+^.

c *M* = 0.77 Ti^4+^ + 0.19 Li^+^.

d The occupancy factor and the atomic displacement parameter were refined independently avoiding their strong correlation. The large atomic displacement parameter may be partly due to wide positional distribution of these guest species.

**Figure 5:**
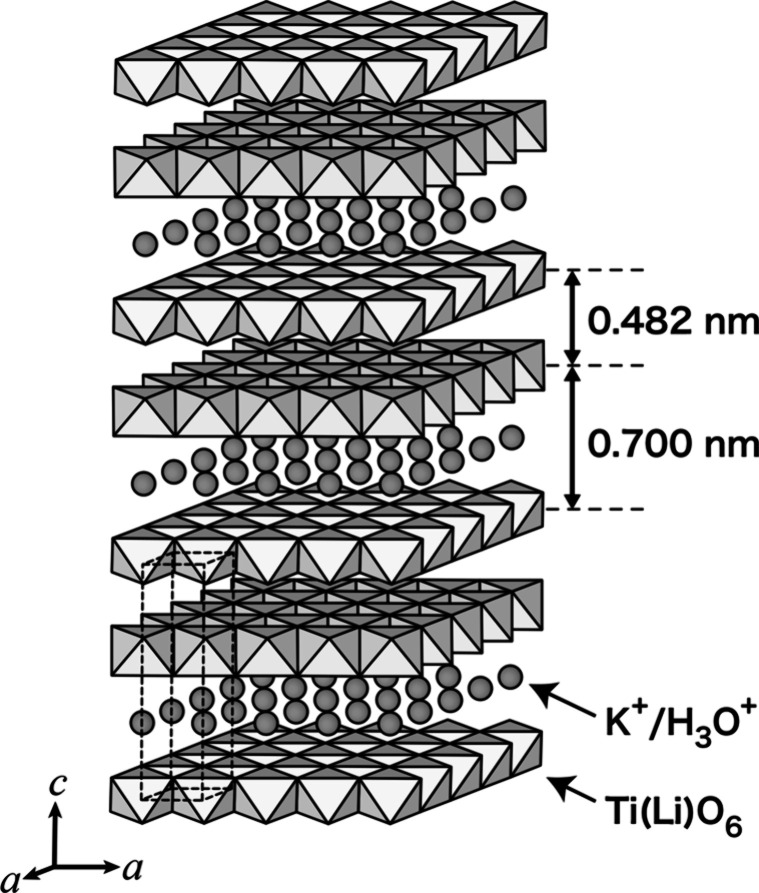
Staging structure of the K^+^ ion-exchanged phase.

The K^+^ ion-exchanged phase (K-NTLO) can be characterized by the so-called staging structure. It is well-known that graphite forms the various staging structures upon intercalation of guests, typically alkali metals.^33–36^ Apart from graphite intercalation compounds, some layered compounds, such as layered double hydroxides^37–40^ and interstratified clay minerals,^41–43^ were reported to evolve such a unique structure. However, the formation of the staging structure in high crystallinity, which allows full structure refinement, is rare. Although there have been a number of studies for a range of layered titanates and their derivatives as described in the Section 1, to the best of our knowledge, this is the first example of the staging-structured titanate. The layer architecture of the α-NaFeO_2_ type is rather thin and flexible, which cannot adequately screen the electrostatic repulsion between guest cations located in neighboring interlayer space. Thus, the staging structure was produced, avoiding the occupancy of K^+^ ions at every interlayer space. The flexible layer is also favorable, stabilizing the staging structure by forming domains similar to graphite known as the Daumas and Hérold model.^44^ Recently, the staging structure was reported in the electrochemical intercalation process of LiCoO_2_, the layer of which is also thin and flexible.^45^ On the other hand, the other titanates are composed of relatively thicker corrugated layers, which can fully screen the electrostatic interaction. The interlayer cations may be considered to be isolated from those in the neighboring gallery.

The narrower interlayer spacing of 0.482 nm can be taken as the thickness of the titanate layers. This value is comparable to the layer thickness of LiCoO_2_ with a similar layer architecture.^46^ The difference of 0.218 nm (= 0.700–0.482) in the gallery heights should be related to the size of K^+^ and H_3_O^+^ ions. It is to be noted that a change in the *c*-parameter for the ion-exchanged phases is rather modest in comparison with a variation of the ionic size for Li^+^ to Cs^+^ (Figure 3). This may indicate that H_3_O^+^ ions play a main role in opening the wider interlayer gallery.

### 3.4 Electrochemical Intercalation/Deintercalation

Previous study showed that NTLO can work as a superior anode material because of negligible volume change upon electrochemical cycling of the intercalation/deintercalation process.^27^ Thus, it is of interest to explore the electrochemical performance of the ion-exchanged derivatives obtained in this study.

The compounds of Na-KNTLO with the staging structure and the pristine NTLO were employed as working electrodes in the coin-type cell filled with 1 M LiPF_6_ in EC/DEC, and their electrochemical intercalation/deintercalation behaviors of Li^+^ ions in lithium half-cells were examined at 50 mA/g in the voltage range of 0.3–3.0 V vs Li counter electrode. As shown in Figure 6, the capacity delivered in the initial Li^+^-ion intercalation process for Na-KNTLO reached ∼210 mAh/g due to the formation of a solid electrolyte interphase (SEI) and then decreased in the following cycles. The relatively large capacity observed for Na-KNTLO may be ascribed to the decomposition of water persisted in the material. After 10 cycles, the reversible capacity of 82 mAh/g was obtained, corresponding to 0.23 Li^+^ insertion per formula unit, which is slightly smaller than the available unoccupied sites (0.25 (= 1 – 0.17 – 0.58)) in Na_0.17_H_0.58_Ti_0.77_Li_0.17_O_2_, assuming that the water has been decomposed. In contrast, the pristine NTLO showed a reversible capacity of 67 mAh/g, which corresponds to 0.22 Li^+^ insertion per formula unit. This value is much smaller than the available vacancy (0.32) in Na_0.68_Ti_0.77_Li_0.23_O_2_. The crystal structure and morphology of the electrode materials that experienced the intercalation/deintercalation of Li^+^ ions were examined. Upon 50 cycles, the 002 reflection shifted to a higher angle (*d* = 0.492 nm) in the XRD pattern for Na-KNTLO, and the peaks due to 001 and 003 reflections disappeared (Figure 7). These changes can be ascribed to the ion exchange of Na^+^ ions with Li^+^ ions in their repeated intercalation/deintercalation processes because of the large excess of Li^+^ in the system. The disappearance of 001 and 003 peaks from Na-KNTLO suggests the intercalation of Li^+^ ions into the empty interlayer galleries during the process, leading to a loss of the staging structure. The shift of the 002 reflection was also observed for the pristine NTLO after repeated intercalation/deintercalation processes, and its *d*-spacing decreased to 0.496 nm, which is close to that of Na-KNTLO after the cycles. On the other hand, the platy morphology of Na-KNTLO and NTLO did not change significantly after 50 cycles of the process, as observed by SEM (Figure S6).

**Figure 6:**
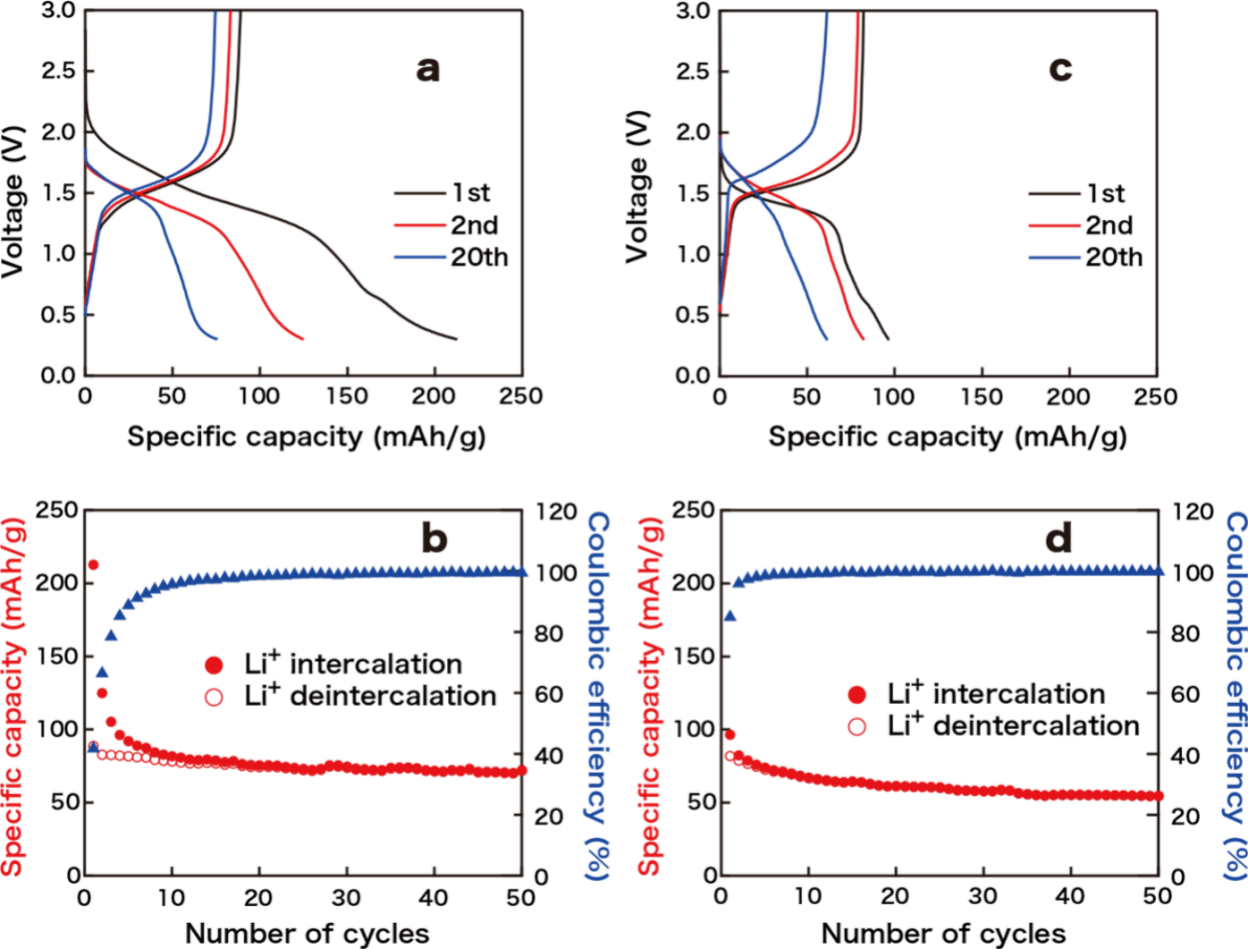
Intercalation/deintercalation curves (first, second, and 20th cycles) of Li^+^-ion batteries using Na-KNTLO (a,b) and pristine NTLO (c,d) and their specific capacity cycle performance at 50 mA/g in the voltage range of 0.3–3.0 V, starting from the intercalation process.

**Figure 7:**
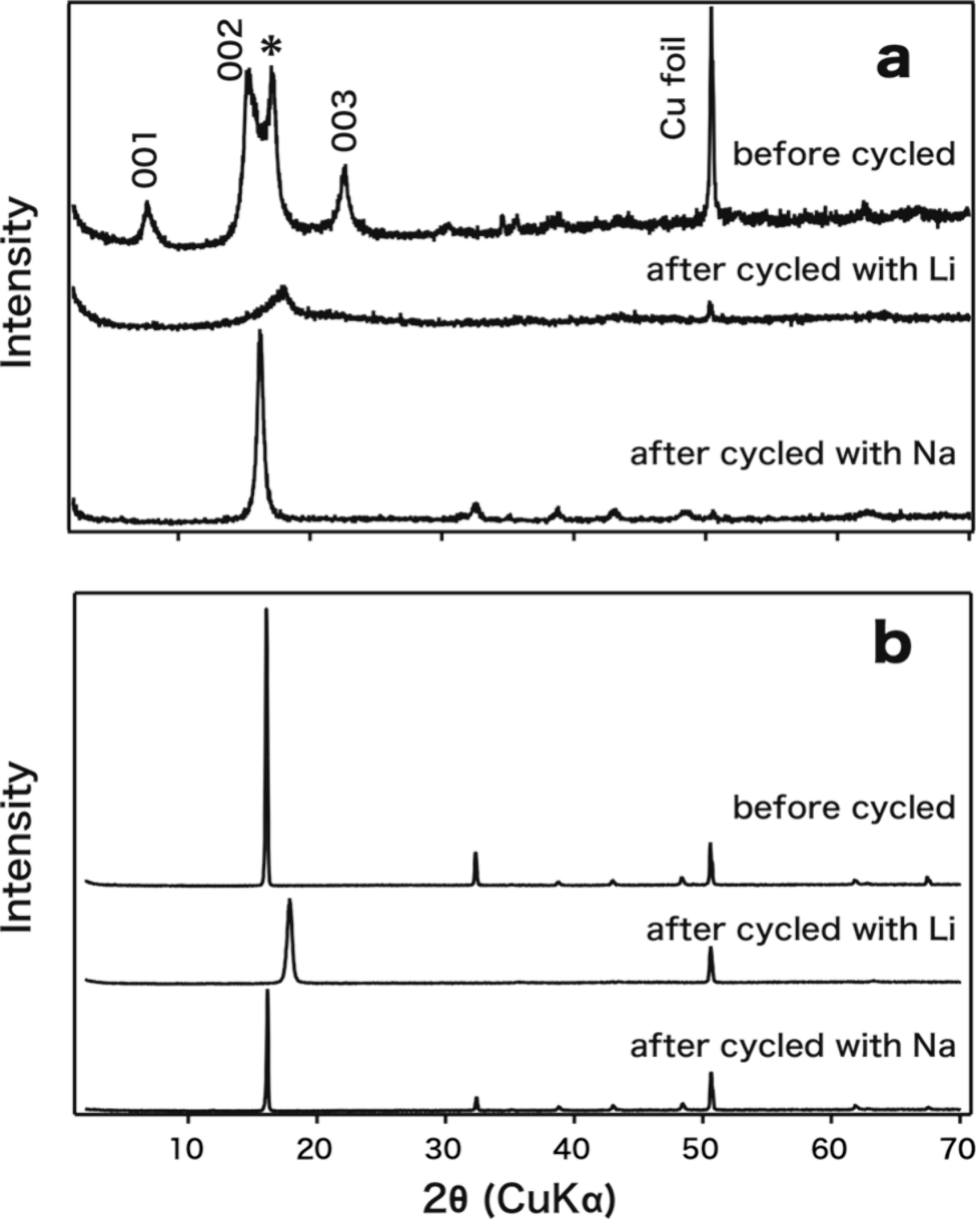
XRD patterns of Na-KNTLO (a) and pristine NTLO (b) before and after intercalation/deintercalation of Li^+^ ions or Na^+^ ions at 50 mA/g in the voltage range of 0.3–3.0 V. The peak designated with an asterisk (*d* = 0.516 nm) may be ascribed to dried Na-KNTLO formed during the electrode preparation.

Intercalation/deintercalation of Na^+^ ions was also studied for Na-KNTLO and NTLO (Figure S7). The reversible capacity obtained after 10-cycle intercalation/deintercalation was 77 mAh/g for Na-KNTLO, corresponding to 0.21 Na^+^ insertion per formula unit, which is slightly smaller than the available vacant sites (0.25) in Na_0.17_H_0.58_Ti_0.77_Li_0.17_O_2_. In contrast, the pristine NTLO showed a reversible capacity of 56 mAh/g, which corresponds to 0.18 Na^+^ insertion per formula unit. This value is much smaller than the available vacancy (0.32) in Na_0.68_Ti_0.77_Li_0.23_O_2_. As shown in Figure 7, the peaks due to 001 and 003 reflections in the XRD pattern for Na-KNTLO disappeared, indicating the loss of the staging structure, as is the case in the intercalation/deintercalation of Li^+^ ions. The 002 reflection from Na-KNTLO was shifted from *d* = 0.577 to *d* = 0.546 nm after the cycles, the latter of which is close to the 002 reflection from NTLO before (*d* = 0.552 nm) and after (*d* = 0.548 nm) intercalation/deintercalation of Na^+^ ions. The repeated intercalation/deintercalation processes might promote the equivalent insertion of Na^+^ ions in each gallery of Na-KNTLO, transforming its crystal structure similar to that of NTLO.

Although the staging structure of Na-KNTLO was lost during cycling, Na-KNTLO showed more efficient utilization (91% and 86%) of available vacant sites compared to the pristine NTLO (68% and 56%) in the reversible intercalation/deintercalation of Li^+^ and Na^+^ ions, respectively. Repeated intercalation/deintercalation into the empty interlayer spaces of the staging structure may facilitate a more efficient utilization of the interlayer galleries, resulting in a higher reversible capacity.

## 4 Conclusions

The α-NaFeO_2_-type layered sodium lithium titanate was synthesized by solid-state calcination for the stoichiometry of Na_*x*_Ti_1–*x*/3_Li_*x*/3_O_2_ of 0.68 ≤ *x* ≤ 0.70. Li^+^, as well as Ti^4+^ ions occupy the octahedral site in the host layer, while Na^+^ ions are accommodated in the trigonal prismatic cavity of the interlayer space. The interlayer Na^+^ ions could be exchanged with alkali metal ions (Li^+^, Na^+^, K^+^, Rb^+^, and Cs^+^) and protons/oxonium ions when brought into contact with aqueous solutions of the corresponding alkali metal salts. The ion-exchange process produced the staging structure, in which the occupied and unoccupied interlayer galleries alternate. The staging-structured alkali titanates were applied as a host material for electrochemical intercalation/deintercalation of Li^+^ and Na^+^ ions, and we found that the specific capacities of Na-KNTLO were higher than the pristine NTLO for both Li^+^ and Na^+^ ions, suggesting superior performance of the staging structure for energy storage.

## Supplementary Material



## Notes

SEM images of as-synthesized NTLO, its ion-exchanged phases, and Na-KNTLO and NTLO before and after Li^+^ ion intercalation/deintercalation; Rietveld fitting of XRD data for NTLO and its structural parameters; XRD data for NTLO treated with LiCl and NaCl; and Na^+^-ion intercalation/deintercalation behaviors for Na-KNTLO and NTLO (PDF)
