# Supplementary material for: Layered Alkali Metal Titanate with the Staging Structure and Superior Electrochemical Performance
Source: Inorg Chem. 2025 Aug 4;64(32):16289–96. doi: 10.1021/acs.inorgchem.5c01712 (PMC12365883; doi:10.1021/acs.inorgchem.5c01712)
Supplement: Supplementary file 1 [file ic5c01712_si_001.pdf]

Layered Alkali Metal Titanate with the Staging Structure and Superior Electrochemical Performance

Aranee Pleng Teepakakorn,[1,2] Tomohiro Tanaka,[1] Nobuyuki Sakai,[1] Yasuo Ebina,[1] Takayuki Kikuchi,[1] Renzhi Ma,[1] Makoto Ogawa,[2] and Takayoshi Sasaki[1,*]

[1] Research Center for Materials Nanoarchitectonics (MANA), National Institute for Materials Science (NIMS), 1-1 Namiki, Tsukuba, Ibaraki 305-0044, Japan

[2] School of Energy Science and Engineering, Vidyasirimedhi Institute of Science and Technology (VISTEC), 555 Moo 1 Payupnai, Wangchan, Rayong 21210, Thailand

*Corresponding author. E-mail: sasaki.takayoshi@nims.go.jp

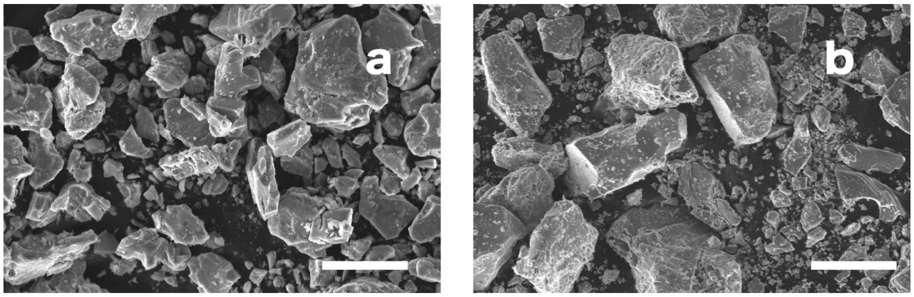

Figure S1. SEM images of the calcined products for $Na_xTi_{1-x/3}Li_{x/3}O_2$ with $x = 0.68$ (a) and 0.70 (b). The scale bar indicates 50 μm.

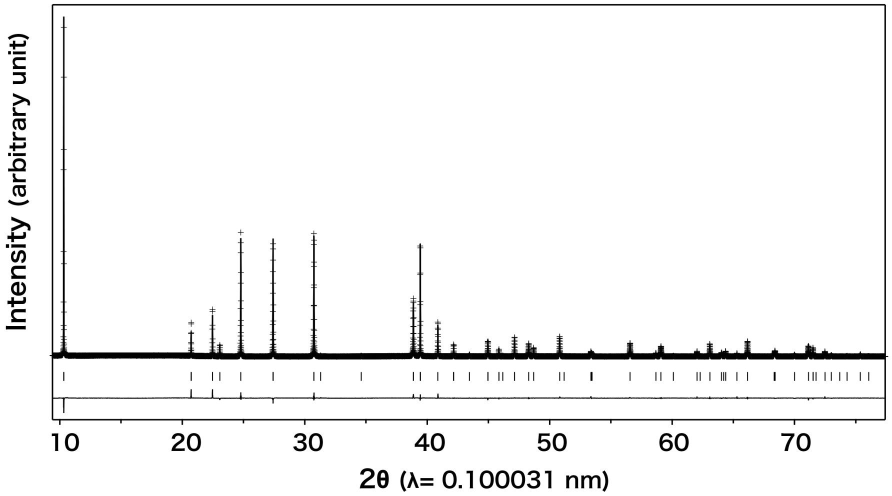

Figure S2. Rietveld fitting of synchrotron X-ray diffraction data for $Na_{0.68}Ti_{0.77}Li_{0.23}O_2$. Observed and calculated profiles are denoted by dotted and solid lines, respectively. The difference between them and locations of reflections are indicated at the bottom.

Table S1. Structural parameters for $Na_{0.68}Ti_{0.77}Li_{0.23}O_2$.

Atom	Position	Occupancy	*x*	*y*	*z*	B_{eq} ($\times 10^{-2}$ nm^2)
Na1	2b	0.245(1)	0	0	1/4	3.21(6)
Na2	2d	0.467(1)	2/3	1/3	1/4	1.76(3)
M *	2a	1	0	0	0	0.017(7)
O	4f	1	1/3	2/3	0.09428(6)	0.27(1)

* M = 0.77 Ti^{4+} + 0.23 Li^{+}

Hexagonal, $P6_3/mmc$ (No. 194), a = 0.296380(3) nm, c = 1.11208(1) nm

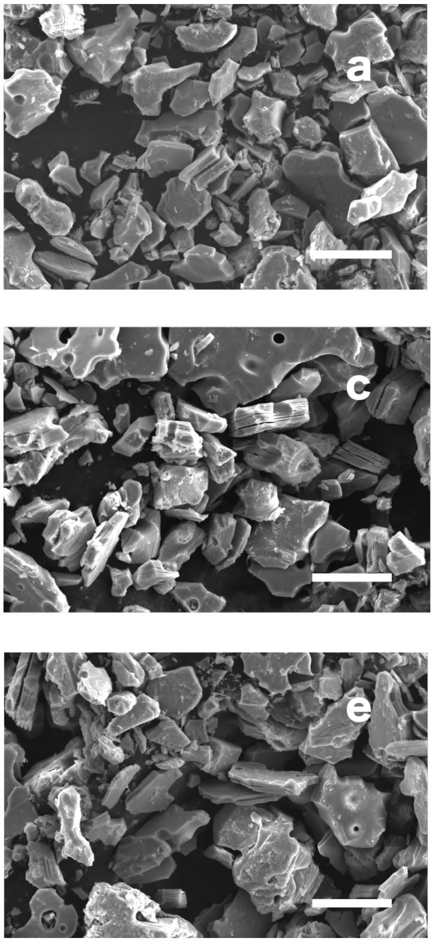

Figure S3. SEM images of the ion-exchanged phases after the treatment with aqueous solutions of LiCl (a), NaCl (b), KCl (c), RbCl (d), and CsCl (e). The scale bar indicates 50 μm.

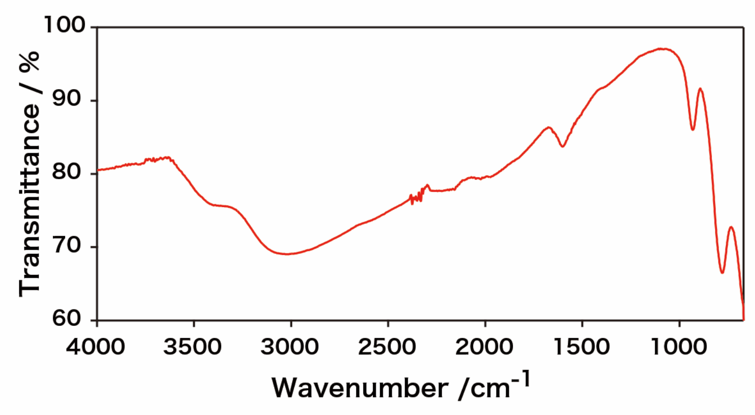

Figure S4. FT-IR spectrum of the ion-exchanged phase (K-NTLO).

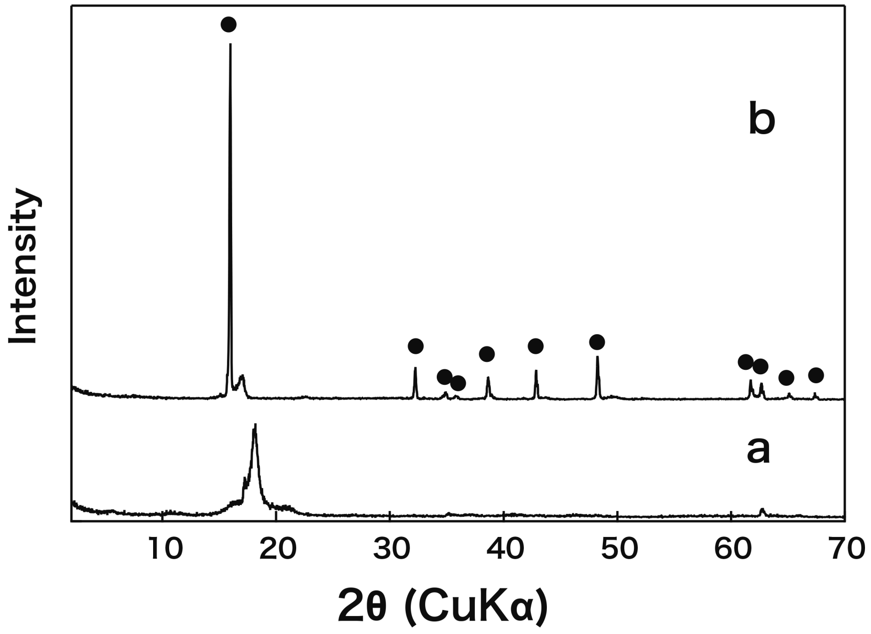

Figure S5. Powder XRD data of NTLO after the treatment with aqueous solutions of LiCl (a) and NaCl (b). Diffraction peaks with circles are attributable to the pristine NTLO.

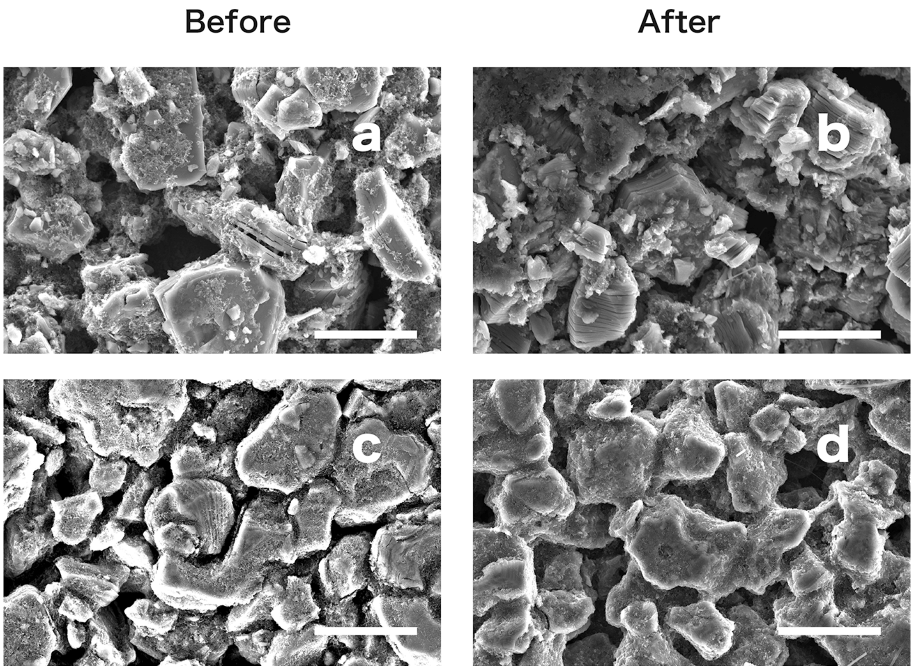

Figure S6. SEM images of Na-KNTLO (a, b) and pristine NTLO (c, d) before and after 50 cycles of intercalation/deintercalation of Li^+ ions at 50 mA/g in the voltage range of 0.3–3.0 V. The scale bar indicates 30 μm.

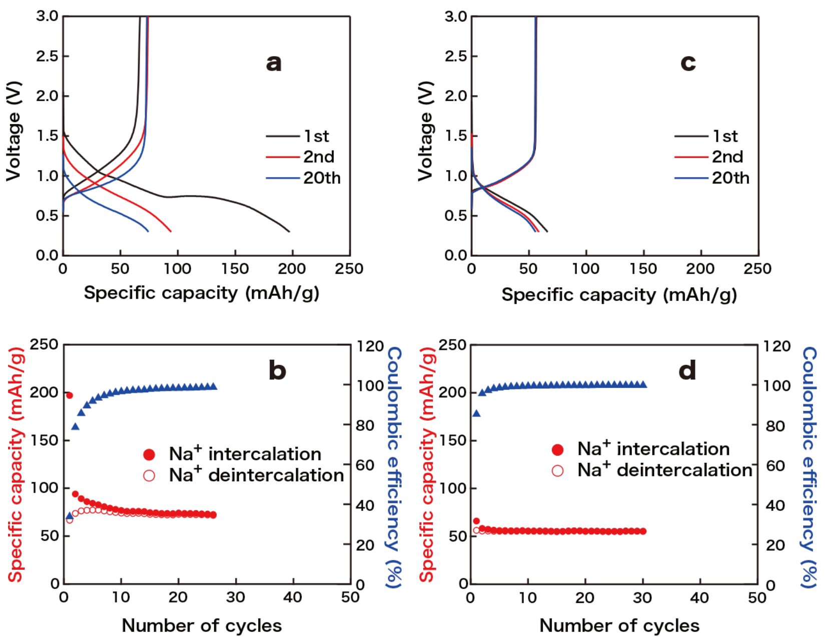

Figure S7. Intercalation/deintercalation curves (1st, 2nd, and 20th cycles) of Na^+ ion batteries using Na-KNTLO (a, b) and pristine NTLO (c, d), and their specific capacity cycle performance at 50 mA/g in the voltage range of 0.3–3.0 V vs Na counter electrode in 1 M NaTFSI EC/DMC, starting from the intercalation process.
